# Advances and Innovation in Acute Type a Aortic Dissection

**DOI:** 10.3390/jcm13247794

**Published:** 2024-12-20

**Authors:** Madison A. Grasty, Kendall Lawrence

**Affiliations:** Division of Cardiovascular Surgery, University of Pennsylvania, Philadelphia, PA 19104, USA

**Keywords:** innovation, aortic dissection, novel therapies, operative management, endovascular technique

## Abstract

The prompt and appropriate management of acute type A aortic dissections is imperative for patient survival. Advances in medical technology have broadened the adjuncts available to treat the spectrum of pathology within this population. The role of medical management prior to surgical intervention and the components of operative management, including cannulation strategies, neuroprotection, and the extent of aortic intervention, have been explored in-depth within the literature. More recent work has identified novel open and endovascular techniques available to treat acute type A dissections. This review aims to summarize the literature, with a particular focus on innovation in cardiac surgery and its role in the care of this high-risk population.

## 1. Introduction

Acute type A aortic dissections (ATAADs) are the most common pathology to affect the aorta and are also the most deadly [1,2]. Historically, mortality has been described as 1–2% per hour in the first 48 h [3]. A tear in the intima of the aorta can create a false lumen and result in insufficient blood flow to end organs, leading to various malperfusion syndromes, significantly increasing the risk of mortality [4]. Thus, prompt management of these patients is imperative to decrease morbidity and mortality. This review will describe retrospective and current outcomes in surgical repair of ATAADs, define the current standard of care for management, and discuss recent technological advances and surgical innovations within the field.

## 2. Medical Management

Historically, patients with ATAADs had staggeringly high mortality rates ranging from 21 to 37%, irrespective of patient age, and exceeding 50% for patients who presented in shock [5,6,7,8]. More recently, the mortality rate from ATAADs has reduced to 5.8% within 48 h after diagnosis [5]. The improved mortality rates can be attributed to earlier diagnosis and standardization of medical and surgical management of these complex patients.

After the diagnosis has been made with a computed tomography (CT) angiogram, anti-impulse therapy should be initiated to stabilize the patient prior to transfer for definitive treatment [9]. When initiated quickly, anti-impulse therapy can improve myocardial perfusion, suppress false lumen propagation, and prevent aortic rupture [9]. Ultimately, definitive treatment is surgical intervention; therefore, timely transfer to an appropriate facility is critical. Recent work has demonstrated that patients with ATAADs have significantly improved survival, with a reduction in operative mortality from 20 to 10%, when transferred to high-volume hospitals with high-volume surgeons, particularly those with expertise in aortic surgery [10,11,12,13,14,15,16,17]. This dramatic reduction in mortality was hypothesized to be due not only to surgeon expertise but also to the presence of a focused knowledgeable team with advanced perioperative care [12]. The significant decline in hospital mortality is one of the many benefits when patients are transferred to high-volume aortic centers. Additionally, stroke rate, bleeding complications, and time to intervention also decrease at aortic centers [12,13]. These improvements are a result of the offerings and treatment strategies of an aortic center. For example, the role of a dedicated aortic operative staff, standardization of operative techniques (i.e., cooling and cannulation strategies), and surgeon/center volume have been identified as imperative components of a patient’s treatment, which each play a role in reductions in patient mortality and morbidity.

## 3. Surgical Management

Emergent surgery remains the standard of care for the management of ATAADs. The most important principles of repair include the identification and resection of the primary entry tear and the restoration of flow to the true lumen. Numerous studies have shown that distal anastomosis ought to be performed under circulatory arrest to ensure adequate inspection of the ascending aorta and provide improved late durability [18].

### 3.1. Cannulation

Selecting the arterial canulation site is a complex decision based on a review of pre-operative CT imaging, surgeon comfort, and patient acuity. Traditionally, femoral cannulation was the preferred cannulation strategy for ATAADs, but this has fallen out of favor due to the retrograde flow and potential to propagate dissections or lead to embolic strokes [19,20,21]. The presumed mechanism is the retrograde arterial flow, which can cause malperfusion and occur concurrently with embolic material flowing to the brain and end organs. Axillary cannulation has now become the preferred site of cannulation for ATAADs at most centers due to its reliability and capacity to offer antegrade cerebral protection, but it can be time consuming in obese or unstable patients and has been associated with ipsilateral strokes [3,21,22,23]. Finally, central or direct aortic cannulation has now become the preferred and standard cannulation strategy at many high-volume centers because of the ease of cannulation and more reliable cerebral perfusion on bypass [24,25,26,27]. Its safety, particularly when performed under image guidance to ensure the aortic cannula is within the true lumen, has been validated [21,22]. Considerations for the cannulation strategy include the degree of aortic calcification, location of the intimal tear, involvement of head vessels, and the patient’s clinical status. However, irrespective of the patient presentation, central cannulation has been proven to be a safe strategy in the perioperative setting and has an equivalent, if not lower risk of complications when compared to axillary or femoral cannulation.

### 3.2. Cerebral Protection

Neuroprotection is a critical component of operative planning in patients with ATAADs. Historically, deep hypothermic circulatory arrest (DHCA) was used without adjunctive cerebral perfusion for the management of ATAADs. A reduction in the cerebral metabolic rate due to deep cooling was thought to be sufficiently protective. Over the last 30 years, however, the consequences of this strategy became more apparent. Patients who underwent cerebral protection with just DHCA alone experienced higher rates of permanent neurologic dysfunction and mortality [28]. More recently, retrograde cerebral perfusion (RCP) or antegrade cerebral perfusion (ACP) has been used with greater frequency as an adjunct during circulatory arrest. A large retrospective analysis of 6387 patients who underwent ATAAD repair found that the use of ACP or RCP was associated with a reduced risk of death or stroke compared to hypothermic circulatory arrest without cerebral perfusion [29]. Additionally, Guo et al., in a meta-analysis of 7023 patients, described a lower incidence of temporary neurological deficits in patients who received DHCA + ACP as opposed to DHCA + RCP. Multiple retrospective studies have compared RCP to ACP and failed to identify a superior cerebral protection strategy during ATAADs repair, but nonetheless, ACP with varying degrees of hypothermia has been used with an increasing frequency for ATAAD repair [28,29,30,31,32,33,34,35,36,37,38,39,40].

Systemic temperature management is an important consideration for intra-operative neuroprotection and remains a fiercely debated topic. Deep hypothermia (<20 °C) has traditionally been used as it has been shown to reduce the metabolic demands of cerebral tissue by decreasing energy consumption and oxygen requirements. More recently, moderate hypothermia (20.1–24 °C) has been used with an increasing frequency during ACP. Proponents argue that moderate, as compared to deep hypothermia, reduces operative times and blood product utilization [41,42,43,44]. A recent randomized control trial found that low–moderate hypothermia was non-inferior to traditional deep hypothermia when examining global cognitive change 4 weeks after aortic surgery, although structured memory was better in the deep group [45]. To date, no randomized trial has been conducted on temperature management in ATAADs. A large retrospective study using the Society of Thoracic Surgeons database identified a slight linear association between lower nadir temperatures and lower odds of mortality and major neurological events [29].

Reliance on DHCA alone is no longer a sufficient strategy to employ cerebral protection during the repair of aortic arch pathology. The utility of RCP or ACP is now considered a necessary adjunct to reduce neurological morbidity after emergent or elective cardiac surgery. Additionally, special attention is required for temperature management. DHCA, despite its impact on metabolic rates, is not as protective as once thought, and the protective effects with moderate instead of deep hypothermia seem to be superior. Irrespective of patient presentation (elective vs. emergent), moderate hypothermia is demonstrated to be safe and reduces the utilization of hospital resources.

### 3.3. Extended Arch Versus Hemiarch

**Figure 1:**
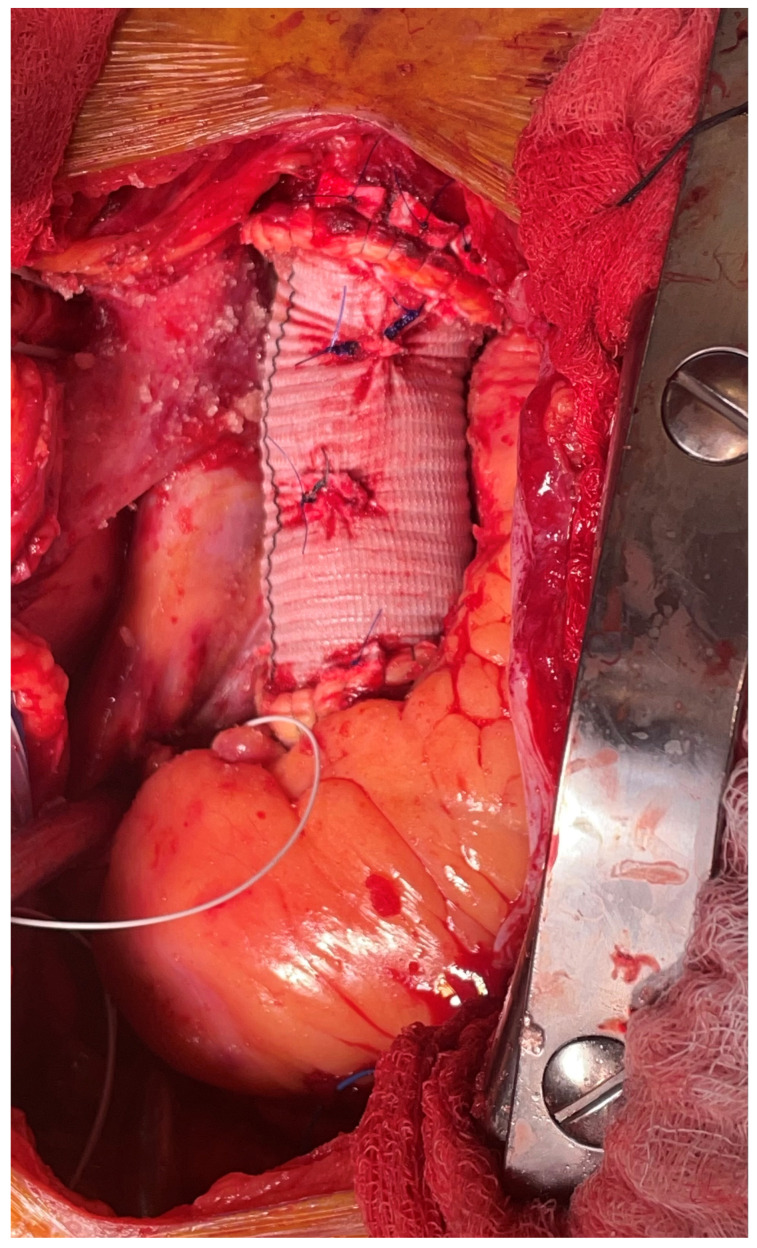
Status post ascending hemiarch placement.

Optimal management of the aortic arch needs to balance the potential benefit to the patient with the added risks [9]. When a hemiarch (Figure 1) is utilized, the distal aorta is not addressed with this management strategy, and the rates of reintervention are as high as 30% in some reports [46]. An extended arch operation may be mandated when there is an arch tear, arch aneurysm, or concern about a heritable thoracic aortopathy [9,47]. Outside of those traditional indications, some advocate for a more extended arch operation upfront to reduce the potential need for future aortic reintervention, promote distal aortic remodeling, and reduce the likelihood of ongoing malperfusion, particularly cerebral malperfusion when head vessels are dissected [48,49,50]. Compared to a hemiarch, a total arch replacement has traditionally been associated with higher mortality and neurologic events, but recent studies at high-volume aortic centers have demonstrated comparable outcomes as institutional experience has increased [51]. A randomized trial is ongoing that is evaluating standard hemiarch versus extended arch repair techniques [52].

### 3.4. New Surgical Techniques

In recent years, many novel surgical techniques have been developed to improve perioperative outcomes, minimize ongoing malperfusion, reduce surgical mortality, and prevent the need for future aortic reintervention. One such innovation, known as the “branch-first” technique, has been designed to mitigate the need for extended periods of circulatory arrest during total arch replacements. This technique utilizes sequential clamping of the head vessels with reconstruction using a specially designed trifurcated graft to allow continuous cerebral perfusion. Matalanis et al. describe a series of 30 patients undergoing repair of type A dissection utilizing this strategy and demonstrate excellent outcomes [53]. In their series, only two patients experienced neurologic dysfunction; 47% were discharged from the ICU within 48 h, and 27% of patients required no blood products. More recently Fleishman et al. published their experience over 4 years with 76 patients who underwent the branch-first technique [54]. Compared to patients undergoing traditional total arch replacement, the branch-first group had lower antegrade cerebral perfusion times and 30-day mortality with a trend toward lower rates of neurologic outcomes [54].

To reduce distal anastomotic reentry tears and potentially reduce the need for future aortic reintervention, the Ascyrus Medical Dissection Stent (AMDS) has also been increasingly utilized at select centers. It is a hybrid device made of a partially covered Teflon graft (proximally) and a cylindrical nitinol frame (distally) that sits in the aortic arch [55]. The nitinol frame in the arch is designed to stabilize the dissection flap, promote false lumen thrombosis and aortic wall remodeling. It also encourages positive remodeling of the diseased aortic arch [55]. Early and midterm results from a prospective, nonrandomized international trial of AMDS implantation have recently been published both in Canada and Europe [55,56]. In the Canadian trial, all 16 patients had an identifiable entry tear, and the device was safely implanted with no device-related reinterventions in all patients. False lumen thrombosis and aortic remodeling occurred in 91.7% of patients. This increase in arch false lumen obliteration in patients who received AMDS compared to the hemiarch alone was significant, but the difference in late aortic intervention rates has not yet been demonstrated. There is a prospective trial currently enrolling in the United States, and long-term follow-up studies are underway to determine the role of this device in ATAADs. The utility of the AMDS graft should be considered as a treatment option for patients who present with an acute type A dissection. Its safety and feasibility have been proven, but in light of recent work that has highlighted the morbidity and mortality associated with open total arch repairs in this population, the ability of the AMDS stent to decrease operative times and the necessity of complex arch repairs should not be overlooked [56].

Another increasingly utilized surgical technique is the branched stented anastomosis frozen elephant trunk repair (B-SAFER) technique first described by Roselli and colleagues to improve the safety and reproducibility of extended arch operations. This technique can be performed in several iterations, but it typically involves endovascular stents deployed in one or all of the head vessels through in situ fenestration in the arch graft. Roselli et al. recently described their experience with 178 patients with aortic dissection repaired via the B-SAFER method. In their series, the left subclavian artery was most commonly stented, followed by the left common carotid artery. When compared to conventional total arch replacement, patients undergoing extended arch operations with the B-SAFER technique had significantly shorter operative times and lower blood product usage; reintervention rates and operative mortality were also better in the B-SAFER group, although this was not statistically significant [57]. While the B-SAFER technique (and its adjuncts) remains fairly new, these early results show promising outcomes [57,58].

**Figure 2:**
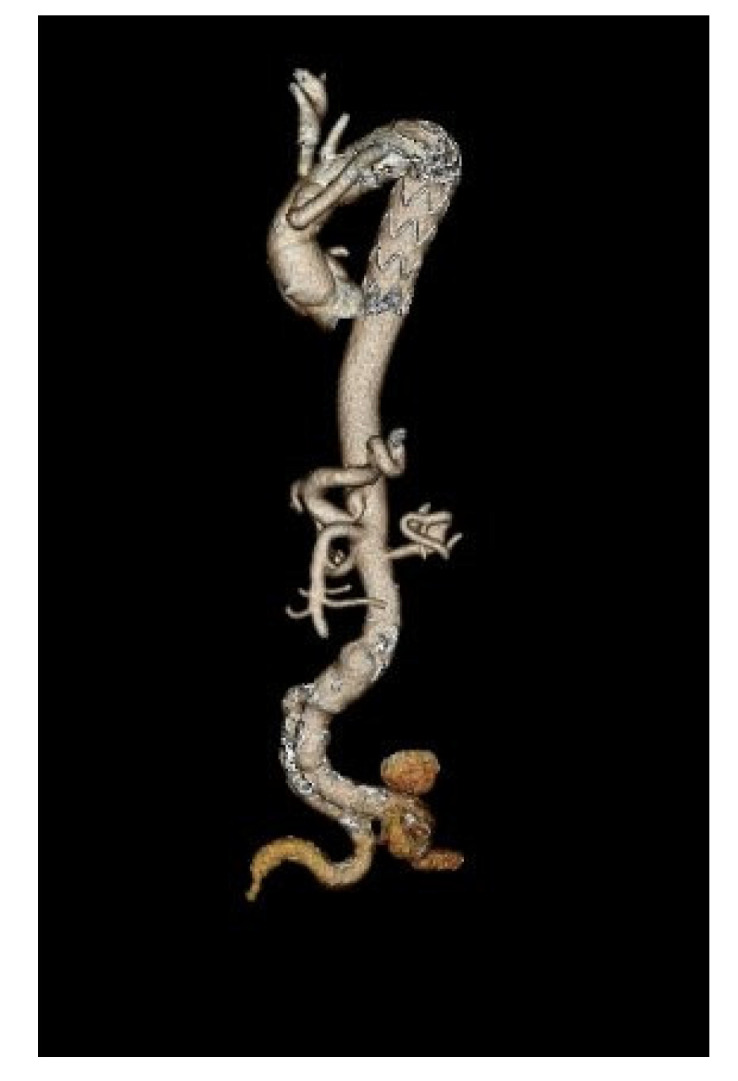
Post-operative computed tomography with 3D reconstruction after Thoraflex placement.

**Figure 3:**
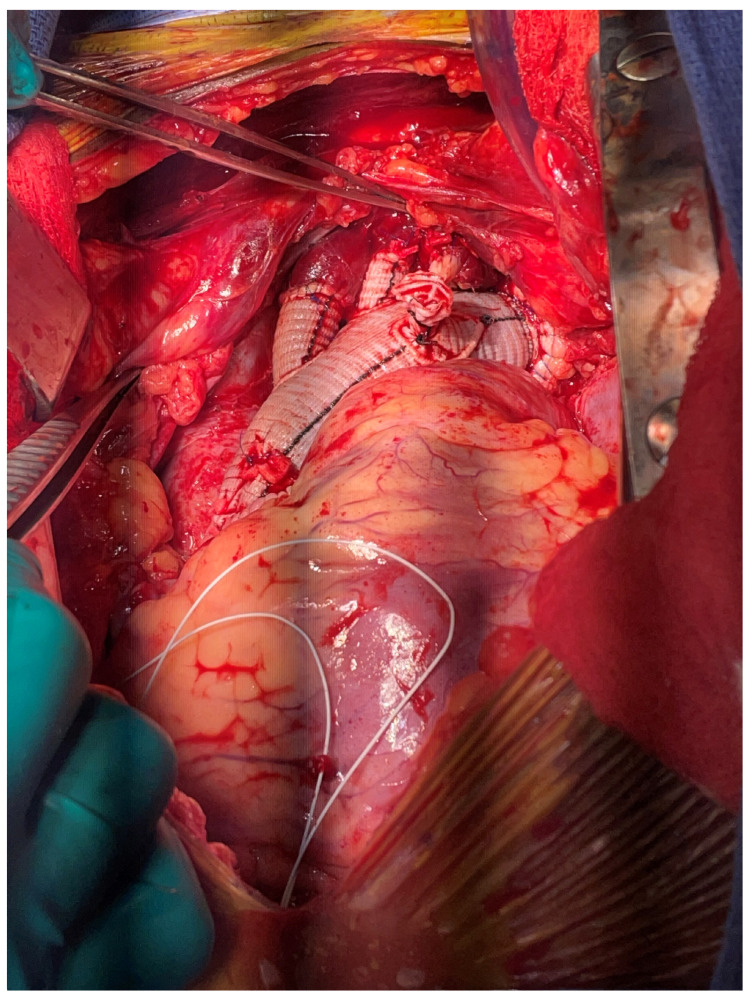
Total aortic arch replacement with frozen elephant trunk.

Finally, the Thoraflex device is another example of recent technology advancing the surgical techniques for ATAADs (Figure 2). This device is a hybrid arch graft proximally with a stent graft distal component. Its design allows for easy deployment of a frozen elephant trunk at the time of total arch replacement (Figure 3). Since its advent, there has been increased usage of frozen elephant trunks. Thoraflex has been shown to be effective in treating acute and chronic dissection pathologies, including those who present with malperfusion syndromes [59,60]. Despite its benefits, Thoraflex does have some drawbacks, including a spinal cord ischemia risk and the common need for subsequent extension in the descending thoracic aorta [61].

### 3.5. Endovascular Advances

Lastly, total endovascular options now exist for ATAAD repair in very select patients, specifically those who were historically deemed to not be surgical candidates due to their risk. The ARISE trial, a multicenter early feasibility study by Roselli et al., introduced a novel device to endovascularly treat AATADs [58]. This landmark trial enrolled nineteen patients; 52.6% had DeBakey type 1, and 84.2% of patients had acute pathology. The mortality rate was 15.8%, with stroke in 5.3% and myocardial infarction in 5.3%. Patients with entry tears that could not be identified or entry tears near the coronary arteries or innominate orifice were excluded, as were patients with severe aortic regurgitation [58]. The ARISE trial demonstrated that an ascending stent graft can be safely utilized to manage zone 0 aortic dissections [58]. Further work with the ascending stent graft is ongoing with the ARISE II trial (Clinical Trial No. NCT05800743). This iteration will evaluate the utility of the ascending stent graft device with a thoracic branch endoprosthesis (TBE) device in a larger subset of patients and is actively enrolling. There is ongoing work to be carried out, but these initial studies have had very promising results in well-selected high-risk patients. This total endovascular treatment option proposes a viable alternative for the most at-risk patients. Historically, they were only offered medical management, which had a mortality rate of 49.7%, but with the TBE device, the ARISE trial described a mortality rate of 15.8%, a significant improvement compared to the alternative. Additionally, in older adults, the rates at which surgical intervention is offered tend to decline, and thus this device provides a suitable treatment option, which is particularly relevant as the population of the United States ages.

More recently, others have expanded the indications for total endovascular repair of ATAADs [62,63,64,65,66,67]. For example, a series with 39 patients examined the total endovascular arch, which contained stent grafts to three branch vessels, through two antegrade and one retrograde branch [62]. The patients included within this study suffered from chronic post-dissection aneurysm or degenerative aneurysms. This multicenter study found that an endovascular strategy was safe (mortality rate 5%) and remained patent at least one year after surgical intervention. In patients with aortic pathology who are found to not be open surgical candidates, the Zenith Ascend TAA Endovascular Graft is a suitable option [64]. A series of 24 patients, examined by Tsilimparis et al., examined elective and emergent cases and found a 79% survival rate at 30 days, with two major strokes and one minor stroke. Within that same period, two patients required reintervention for a pseudoaneurysm and aortic insufficiency, but over the follow-up period, only one patient required reintervention for an endoleak. This work demonstrated that endovascular repair is a necessary treatment adjunct that is safe in addressing various aortic arch pathologies. In addition to these series describing the outcomes of endovascular therapies, case reports have described the utilization of other therapies. The Endo-Bentall procedure was first reported in 2020. The device consists of a self-expanding transcatheter aortic valve and thoracic endovascular aortic repair (TEVAR) with fenestrations for coronary artery stenting, which is deployed through percutaneous femoral access. Their early results demonstrated the technical feasibility in these well-selected patients. Gaia et al. utilized the Endo-Bentall in a patient who had undergone surgical aortic valve replacement in the setting of severe aortic stenosis. The patient initially did well, with a resolution of symptoms, but ultimately, she developed a pseudoaneurysm in her ascending aorta suture line, which fistulized to the skin [68]. She was not felt to be a suitable operative candidate for open repair and thus the patient ultimately underwent an Endo-Bentall procedure. The patient tolerated the procedure well with no endoleaks and was discharged home on post-operative day 7. Additionally, a triple-branched aortic arch endograft that had been customized for another patient was utilized in an emergent setting in a patient with a contained rupture of a descending thoracic aneurysm who failed open operative repair [63]. To customize this device to this patient’s anatomy, the branches of the device were reassigned to their target supra-aortic vessels. The patient survived the procedure, with the completion angiogram showing occlusion of the false lumen and patency of the branches. Follow-up imaging was without endoleak and confirmed false lumen occlusion. Lastly, the Relay Branch device or double inner branch stent graft was utilized as an endovascular treatment option in patients unsuitable for open repair [67]. This system is unique as it provides a solution for supra-aortic vessels with its two inner tunnels, which connect to the innominate artery and left common carotid artery. In Italy, the results of administering this system in an all-male cohort of 24 patients were a perioperative mortality rate of 16.7% and strokes in 12.5% [66]. There were no type I or III endoleaks and no need for aortic-related reinterventions in the follow-up period. These series, while viable, are not without their difficulties and require individual patient consideration. While customization of devices is an option when sufficient time is available, in an emergent setting, physician-modified device reconfiguration may be required by the attending surgeon and requires an additional level of expertise, meaning this may not be a viable option for those unfamiliar with the devices or their nuances [65,69]. Customized devices can take up to two months to produce; however, the work by Wen et al. demonstrates that it is not only safe but also provides a durable repair when no alternative exists [69]. Additionally, attention to the coronary ostia and the sizes of devices that can be utilized based on the patient anatomy are important operative considerations. Lastly, there is no standardized anticoagulation regimen for this population, and if a patient has contraindications for anticoagulation, then endovascular devices are potentially not an option.

This work demonstrates the need for and utility of total endovascular aortic repair options. These permit the treatment of patients in whom surgical options were deemed prohibitive and thus allows for the treatment of a broader scope of patients.

## 4. Conclusions

AATAD is a pathology with a mortality rate of up to 60% without surgical intervention. In the last 50 years, there have been significant advances in medical and surgical management, which have contributed to significant reductions in patient mortality. Most recently, there have been a number of both open and endovascular techniques developed, which will likely further contribute to reductions in morbidity and mortality.
